# American Dental Association

**Published:** 1885-11

**Authors:** 


### THE DENTAL REGISTER.

Vol. XXXIX.] NOVEMBER, 1885. [No. 11.

### PROCEEDINGS.

### American Dental Association—Twenty-Fifth Annual Meeting.

REPORTED BY MRS. M. W. J.

*Continued from October.*

THURSDAY, 9 A. M.

The president, Dr. J. N. Crouse, in the chair.

The secretary read a letter from Dr. Geo. A. Mills, Brooklyn, for twenty-one years a member of the Association, tendering his resignation. Dr. Mills said that the Association had done much for him, while he had tried to do something for it; that he was full of hope and confidence in its ultimate attainments.

The special committee on the question of appropriations to the sections for the encouragement of original scientific research, reported through the chairman, Dr. Peirce, of Philadelphia. After referring to the scientific investigations carried on in other countries, as in Germany, under Government patronage, or in England, under appropriations from the British Scientific Association and other Societies, and the immense results accomplished compared with the meagre and incomplete results of individual effort—the prestige of the American Dental Association being compromised by lack of originality in papers and reports—the committee recommended that appropriations be made to different sections for the promotion of original investigation; the money to be expended in the purchase of instruments, materials and books, in the employment of experts in the different sciences, artists, copyists, for expenses of printing, of engraving, traveling, etc.; the things that money could do in furthering this great work being almost limitless.

Report open to discussion.

Dr. W. H. Morgan said, that when the subject was first brought before the Association he had announced himself in favor of the committee, but opposed to the appropriation. That since thinking the matter over, and especially while listening to the valuable paper of Dr. Ingersoll, and learning that he had traveled a thousand miles, and spent much time in pursuit of these investigations, comparing the results with those of other men, he had come to the conclusion that, though this might be a work of love with such men as Dr. Ingersoll, yet that men who were able and disposed to do that kind of work ought to at least have their traveling expenses paid. He was therefore ready to vote for the appropriation.

Drs. H. A. Smith, and C. W. Spalding objected to the insignificant amount suggested in the report, which would go but a very little way in the purchase of type-writers, microscopes, and the delicate and costly instruments required in accurate scientific investigations.

Dr. A. W. Harlan thought that such appropriations would soon require assessments and eventually break up the Society.

After some further discussion the report was adopted, and the sum of $200 each appropriated to Sections V, VI, and VII.

Discussion of the subjects suggested by Section IV.

Dr. Kulp spoke of the different methods of attaching crowns to roots, and the great improvements made over the old pivot-crowns. He said that he had recently seen a crown which, although a patented article, he was happy to place before the Association, as being, in his judgment, better, in some respects, than anything he had seen. It was almost impossible to withdraw it when once in position, the root was thoroughly sealed and the whole thing perfectly clean. This was the crown of Dr. Low, of Chicago. He said that he had outgrown his old prejudice against patents, from the fact that original inventors, who gave away their best ideas, were so often obliged afterwards to pay royalty on what they had freely given to the profession; that he now advocated patents to protect generous men in the use of their own inventions, and that he would take a good thing wherever he found it, whether in highways or byways. If a thing was good, its origin was immaterial.

Dr. E. Parmly Brown said there were two objections to the Low Crown, the greatest being that the tip of the crown fitting inside of the root, and the latter having to be reamed out, when the root was small, as in labials, it would have a very thin shell, liable to split. He wished to present a crown which he had just perfected in connection with bridgework. That most crowns were weakened by being too much hollowed out, the strain on a tooth was not equal, the lateral strain being insignificant compared with the strain in biting. He had consequently invented a broad flattened pin which gave added strength where the leverage was greatest. His crowns were also rapidly inserted. He had put in three between nine and one o'clock for a lady who was a professor of vocal music and could only give him those four hours for the entire operation.

Dr. Kulp said that the objection raised to the Low Crown by Dr. Brown would be self-evident but for the fact that it had a cap or band which encircled the root, preventing splitting, and forming a concave cup which received the convex end of the crown, securing perfect adaptation.

Dr. Stockton, New Jersey, said there was no subject now engaging the attention of the profession so much as that of bridgework. That it pertained as closely to mechanical as to operative dentistry, and that he had hoped it would have been given to the former section. As it had been included under operative dentistry he had a case he would like to report. There were in the lower jaw the six front teeth, one right bicuspid, and two wisdom teeth. In the upper jaw, the six front teeth and two wisdom teeth. The six upper front teeth were entirely covered and hidden by the lower incisors in occlusion, presenting a very unsightly appearance. Several attempts had been made to supply artificial dentures, without success. He had placed gold caps over the wisdom teeth, throwing the jaws apart so as to leave the upper incisors only slightly behind the lower, filling in the spaces with bridgework. The operation was thoroughly satisfactory, the patient pronouncing it a blessing beyond price.

Dr. E. Parmly Brown said that the Herbst method of filling might be good, but was not the best. It was going back to old styles and necessitated the cutting away of good material in order to make simple cavities. That no man would dare to say that Marshall H. Webb's method of preparing cavities was not correct.

Dr. Patterson, Kansas City, said he thought the Herbst method would prove unreliable. That he had experimented with it for two years, both at the bench and in the mouth. That he could fill the same cavities more rapidly, and more thoroughly with annealed soft gold, or cylinders. He thought the fillings made at the clinic, yesterday, would fail within six months.

Dr. Daboll, Buffalo, said that the best point in the Herbst method was the use of the matrix. That this insured ease and certainty, and saves time and labor; that it made a simple crown-cavity of the most difficult posterior proximal cavities in molars and bicuspids.

Dr. W. A. Spaulding, Minneapolis, said that the Herbst method was good in some places, and in some cavities, but he noticed that its most enthusiastic advocates finished their work with the electric mallet. The one point was to put the gold in solid, no matter by what method, whether Herbst's or Smith's or Brown's. Do what is best for the case in hand. One says he *always* uses the mallet; another *always* uses the matrix; but there are cases where neither are available. Each man should use his own method, and exercise his own judgment.

Dr. W. C. Barrett said that it was not individual cases, but basal principles that should be discussed. There were three principles involved in filling with gold. One was the wedging principle, in use forty years ago, requiring non-cohesive gold—one piece sliding past the other, held in place on the mechanical principle of the wedge. By this system good work was done in saving teeth. Then came cohesive gold, impacted by blows, starting from a single point where it was securely anchored, and built up piece by piece, making a solid homogeneous mass, restoring nature's contour. These two methods involved diametrically opposite principles. In one the gold must slide ; in the other it must not. In one it was wedged, in the other it was welded.

The Herbst method is entirely different in principle; the gold being burnished down; but each of the three methods has its advantages and its merits, and each has something lacking. By the first method tooth form cannot be restored ; by the second gold cannot be retained in contact with a smooth wall. To produce absolute contact the gold must not be impacted, or driven with sharp points; it must be spread out and worked down smooth.

The Herbst system of rotary burnishing produces the most absolute contact with the walls of the cavity, but to build up a solid, homogeneous mass, requires the mallet. To produce finished, complete work, requires the mastery of all systems. Use the Herbst method to insure contact with the walls of the cavity ; use the mallet to build up the mass and condense the surface ; then you will have a perfect operation. The operator who excludes all but his own way—who says "my way is always the right way, and the only right way, and all others are always wrong" will not succeed, will not be a good teacher. We must select that way which is the best for each individual case.

Dr. E. Parmly Brown said that wherever gold was put in human teeth, and needed there, it was needed absolutely solid; that he did not believe the Herbst, or rotary process would put gold in any angle or pit, that he had challenged Herbst to meet him, either in Europe or in America; he had been asked to fill gold tubes as a test but that would not prove anything, and that was not his business; gold could doubtless be worked against a smooth glass surface with the rotary motion, but that was no proof of what it would do in a cavity in preserving the tooth.

Dr. M. F. Rhein, New York, said that Dr. Barrett had struck the key note in regard to combining different methods according to the case in hand; that the proper kind of gold—the peculiar German gold with which to work on the German plan—had only been very recently introduced into this country; that the Herbst method could only be successfully employed with gold which was made especially for that process—a very soft, non-cohesive gold, which was like working the burnisher in a roll of butter—absolutely non-cohesive in the bottle, although as solid in the tooth as if malleted; that he did not consider his clinic as a fair test, as he had worked with borrowed instruments; that up to the point where he used the mallet none of his gold had been annealed, and that the best operators had examined it and pronounced it thoroughly welded; that the failure of work done two or three years ago, claimed as done by the Herbst method, was due, not to the method, but to the use of ordinary gold; work which he had done last September, by the Herbst method, with the German gold, was as perfect as if done yesterday; that he was a pupil of Dr. Webb in the method of preparing a cavity, and contouring, but for the Herbst method no retaining pits or painful undercuts were required, this being one of the great advantages of the Herbst method. For finishing the surface, and for contouring he used the mallet, combining the two methods—annealing the gold very slightly for the mallet. He said that Herbst had shown the greatest ingenuity in the adaptation of the matrix in the anterior teeth, making proximal cavities as simple as molar crown cavities; that it was just as necessary to have good strong walls as if all the work was to be malleted. In very large cavities the walls could be lined with gold by the Herbst method, the centre filled in with oxyphosphate, in which gold is again anchored and malleted for the masticating surface; that there was not the slightest necessity for impacting a large amount of gold if the enamel edges are perfectly sealed and leakage from the exterior prevented. The quantity of gold impacted is not a material point, so that the tooth is preserved.

Dr. Spaulding, Minneapolis, said that he understood the Herbst method to mean the introduction of a new gold—a very pure gold, and only that kind of gold; that he used Hood and Reynold's gold; had laid one piece on another, without annealing, and burnished it together so that it could not be picked apart; any kind of gold that would cohere solidly was good gold; whether the work was done by the Herbst method or by any other method—with a peculiar kind of gold or with any other kind of gold—the point was simply to have all moisture excluded. It was narrow-minded to feel restricted to the use of any one method, or any one material

Dr. Ingersoll, Keokuk, said there were two or three principles involved. That the surfaces brought into contact by burnishing were held together by atmospheric pressure; that when they adhered from the effect of rapid rotary pressure, it was from electrical attraction, which developed cohesion. By this means, taking advantage of these two principles, Dr. Herbst himself can restore contour by his system.

Dr. Atkinson. R. S. Williams makes a foil, of gold upon platinum and iridium. This foil makes a harder surface for mastication than any other known, comparing with ordinary soft gold as steel with soft iron; it can be used in Nos. 120 or 60, or 30. Its great advantage is its hardness; also its near approach to tooth-color. It adheres to ordinary gold better than to tin. Platinum and Iridium, with a large proportion of 120 gold foil, makes excellent plate for clasps—of 24 American gauge. To make a uniform plate, flow melted gold scraps on the platinum and roll—if heated it will make nodules; tap together and run through the mill. Anneal in using, and use hot from the lamp. Williams' gold and platinum is in cylinders, and in foil, and makes a soft gold of good color; adding iridium give a good hard masticating surface.

Dr. Darby, Philadelphia, would ask Dr. Atkinson if he uses 60 and 120 foil as a rule?

Dr. Atkinson. I don't care whether it is 60 or 120, or 420! I cut it into strips, anneal, and carry home.

Dr. Allport, Chicago. While others are speaking, many things pass through my mind that might be useful to younger members. In listening to the discussions regarding the different methods of using gold, and different manners of operating, I am reminded of the saying of an eminent Divine: "There is no heresy so false that it has not in it a germ of truth." It is equally true that there is no doctrine so true that it has not in it something false. One speaker insists that a hard and solid filling is absolutely essential. If the filling is so packed against the wall of the tooth as to exclude moisture and resist mastication, that is all that is necessary — going beyond that is going beyond the point of utility; the additional hardness is a damage to the tooth, being unyielding to thermal changes. At the meeting of the Association in Boston, I called attention to the difference between cohesion and non-cohesion. Non-cohesive gold simply slides on the surface without clogging. No man here will say that the old-fashioned operators did not save as large a proportion of the teeth they filled, as are saved in proportion to-day, or that any more beautiful operations are made now than were made then. Filling and saving a tooth is one thing, building up a tooth is another. There is nothing better for filling and saving teeth than non-cohesive gold, as I said a year ago. I can see much good, and much evil in the Herbst method. It offers much that is attractive; in some ways it may be good, but not for practical, everyday work, and I predict great evil if it is generally adopted. I judge from what I have heard, and from what I have seen; I have not tried it myself. I do not dispute that it may be burnished against the walls, but not into undercuts. How are you going to use straight instruments in uneven cavities? You must cut away and destroy tooth-substance. No man has the right to destroy, where he can save. The new gold may be the best ever seen, but the method of using it is not good; that is, in my opinion. If you use the Herbst method at all, use it in open cavities, of easy access. If I were to use it at all, I should reverse the order and fill the cavity with the mallet, and finish with the burnisher, and for this I would use Abbott's bulbs.

Dr. Thomas, Detroit. We all seek the same end by different roads. There are thorns and thistles which are easy to uproot, if handled in the right way, but some men cling to their own narrow conceptions. There is a great difference between making beautiful fillings and being a good dentist. Fillings may be beautiful to the eye, and yet not save the teeth. When one method is used exclusively, there is danger of carrying it too far. It is due to the patient to produce good results. It must be remembered that we are dealing with a frail, sensitive structure.

There is more danger to the teeth from over-operating than from not enough. Young men were urged to use the matrix on all occasions, but good results can be produced without it. All students were once taught to use Vulcanite rubber, and now few can use anything else. Learn first to make good gold fillings without a matrix; otherwise there will be trouble in cases where a matrix cannot be used.

Dr. McKellops said he had listened attentively to Drs. Allport and Thomas. He would ask Dr. Allport what he called cohesive and non-cohesive? What he used the lamp for, when using the mallet? He had noticed that in using soft foil, Dr. A. took a No. 4 roll in his fingers and stuck it in the flame to make the point stick. Did not that make it cohesive? He liked men to practice what they preached. About the matrix, there was no instrument more useful than those of Dr. Jack; most essential when it was important to save time, or where we can't get up to the cervical wall. Young men must be taught to use it in the place where it would accomplish good work. Referring to what Dr. Atkinson had said of iridium-gold, Dr. McKellops said that he had not had as much experience with it as with platinum-gold, but the experience he had was not satisfactory. It was too stiff; would not yield, or work under the lamp as he wished; that he got better results with No. 60 platinum-gold rolled; he had found nothing equal to it for the masticating surface. The first he had seen was from Blake, of California, in 1876. He had been using it ever since; could produce almost any shade he wanted for contouring front teeth; by taking proper pains, he could get the very best results; across the table it could hardly be distinguished that the teeth were filled. He used the shades 1, 2 and 3, and nothing less than No. 60, Williams', of New York, in light pellets.

Dr. Abbott wished to say one word more with reference to the Herbst method, as he had had a little experience. The *Independent Practitionr* had published illustrations of his set of instruments; that it was impossible to get into all cavities with straight instruments, while the proper curve would admit of doing a hundred things that could not otherwise be accomplished.

The object of his new curved shapes was to get into undercuts. That it was not safe to depend entirely upon the Herbst method. That the rubbing process without annealing would not give sufficient cohesion to resist where there was any purchase. A filling made with the electric mallet, and annealed red-hot all the way through, could not break apart. If it would admit of taking out the matrix, it would stand any use. He remarked that nothing had been said in the discussion of the bridgework of Dr. Parmly Brown; that he was well-pleased with the specimens he had seen, both in the hand and the mouth. It was the finest he had yet seen, admitting of close adaptation to the gum all around, creating no irritation; also, admitting of restoring the perfect shape of the teeth in the mouth, a great advantage in talking.

Dr. Morgan, Nashville, said there was one absolute essential to success in the insertion of artificial crowns, and that was the proper medications and treatment of the root. Success depends more upon the healthy condition of the root than upon the manipulation of the crown. It is well to lay especial stress upon that point, for he had removed beautiful crowns that had been built upon teeth of which the pulp-chamber had never been opened; of course, periostitis had followed. Another point in regard to impacting gold: every angle made in the foil, previous to placing it in the cavity, requires that much more pressure in compacting. If there are a hundred angles in the pellet, it will require a pressure of one pound to flatten it out; if there are a thousand angles it will require ten pounds pressure. Gold in the best form has smooth surfaces; not crumpled up with a thousand angles to break down. Some one said that a filling should be perfectly solid, but there are cases when to be solidly compact, throughout, is a disadvantage; where there is no room for expansion thermal changes will break down frail edges. Another point: hundreds of teeth are saved now that the fathers would have condemned to the forceps; they are saved by our new methods and new materials. Would use matrices in certain localities, but not everywhere; it was said that the matrix made simple cavities in all cases, but this would not always be an advantage, a compound filling being sometimes easier than a simple one.

Dr. Allport. I desire to make an explanation. My friend McKellops said that I annealed my gold and used the mallet, and *therefore* I used cohesive gold. I desire to say that the large part of gold that I use in the filling of a cavity is non-cohesive; when I build up outside of a cavity, I use, as all dentists do, cohesive gold. Now, I will explain how I use gold, so that no one need misunderstand me. Suppose I make a retaining point, which I seldom do, but which those who use cohesive gold are usually obliged to do, no man can fill the retaining point as perfectly with cohesive gold as with non-cohesive gold. I do it in this way: I take a little pellet and anneal a small point, then I put the non-cohesive portion down into my retaining point and fill it perfectly, leaving the cohesive portion on the surface, then take another piece in the same way, putting the non-cohesive point of the second pellet on the cohesive point of the first every time. With this little point of cohesion there is no danger of the second piece tumbling off or rolling about in the cavity, and that is all there is about it. The large majority of my filling is therefore of non-cohesive gold. My friend seems to think that because I use the mallet I must use exclusively cohesive gold. In this he is entirely mistaken, for I introduce the gold in nearly all my operations with mallet force; I only use hand instruments and hand pressure in such locations as it is difficult to make perfect operations with mallet instruments.

Dr. Kulp. If mixed subjects have a similar effect to mixed drinks we shall be badly off for I am going back to bridge work again. It is an old saying, that "the proof of the pudding is in the eating;" it is the same with bridgework. I am a wearer of it myself, and speak whereof I know. I had the misfortune to lose a molar and a bicuspid, and tried twenty different plates and appliances. Later on I lost a lower molar on the other side. I could not masticate on one side with my artificial appliance, nor on the other side because of the lost molar; the other molars began to abrade and the front teeth to push forward and become sore. My attention was called to what we call a quack advertisement of bridgework. I concluded to beard the lion in his den. I wanted my jaws separated somewhat to save the front teeth.

I had the bicuspid and molar capped and a bridge put in to supply the lost teeth. On the other side I had the molar capped and the inner cusp of the bicuspid taken off. My powers of mastication are fully restored, and I have worn the work for two years with perfect satisfaction and feel that I am perfectly restored. When the front teeth are abraded, separate the jaws with caps.

Dr. J. R. Patrick. I hold it man's first duty to know what the material is that he is using. We hear the terms adhesive, and cohesive, and non-cohesive, which creates some confusion. All gold is cohesive if good, where there is nothing to interfere with one molecule coming in absolute contact with another. There are many different names for gold, but they are merely commercial names. One molecule interlocks with another and becomes homogeneous; when non-adhesive something intervenes and prevents the unity of molecules. Cohesive seems to mean adhesive, brought together, and held together like two pieces of wood glued with mucilage. We have spent much time in discussing the Herbst method. It serves a purpose and teaches us how to do better. I can't see how we can get cohesive results as well as by the direct blow. If gold was of the same character as butter it might be done. We must have direct percussion to drive molecules into each other.

Dr. Dyer, Chicago. Gold is cohesive when absolutely pure; passing it through the lamp makes it pure by destroying any film of extraneous matter and makes it cohesive also. Adhesive. ness means adhering to same foreign body; cohesive is where the particles of gold unite when brought together. Cohesive gold is very soft, but so-called "soft gold" is not as soft as cohesive gold when passed through the lamp.

Dr. Will Ames, Chicago, said that he was an advocate of eclecticism in practice, but as to starting a filling on the Herbst method and finishing with the mallet, he could not see the consistency of putting a soft mass in the bottom and using the mallet to condense the surface. By using non-cohesive gold by hand or mallet pressure, we get a solid foundation on which to use the electric mallet for finishing the surface. What objection is there to the mallet? Why depend on welding? What difference does it make, provided that it is made solid? When should we use the mallet and when not? In putting gold in accessible points he was in favor of hand pressure as giving better command of the instrument whether using cohesive or non-cohesive gold. Whether foreign substances rendered it cohesive or not he did not think that any detriment.

Dr. Taft said that Herbst did not stand alone in his method. In 1884, at the International Medical Congress, in London, in the dental section he saw specimens of cavities lined by Dr. Blount in the same manner, with a smooth pointed instrument. It was like pasting gold upon a smooth surface. The smooth walls were lined with non-cohesive gold in this manner and the contour finished with cohesive gold; the lining was very perfectly done and was much admired. The advantage claimed was the thorough adaptation to the walls; better than by any other method. No one could doubt after seeing the specimens and witnessing the operation that it possesses some advantages over all other methods. The principle of the Herbst method is that of a lining all over the walls, with soft foil, introduced with smooth points, with a rotary motion: the center can then be filled with non-cohesive gold. As to the use of the matrix, Dr. Daboll says that all young men should use the matrix in every case of proximal fillings. This must be taken with considerable caution, as it is liable to mislead. Dr. Daboll said that the use of the matrix reduced all proximal cavities to simple crown cavities. There are some points in filling with the matrix which are not common to crown cavities, as in the adaptation to cervical edges, and to the lateral borders all the way up. Where the matrix comes up square against the cervical border there will be a deficiency at that point unless the greatest skill is exercised and the very finest instruments used; it requires more skill than is possessed except by the very few. He would not advise the use of the matrix except where absolutely essential. The matrix will not fit up to curved cervical borders. Dr. Patrick has said no man ought to use a thing that he did not know all about. If that was the case we must all go to school.

Dr. C. W. Spalding wished to ask one question. Dr. Taft had said *soft foil* and then corrected himself, saying non-cohesive. He would like that point explained.

Dr. Taft. The terms soft and non-cohesive are carelessly used interchangeably. Sometimes gold is quite soft and plastic; it will weld. All depends on the management of the gold in its preparation. Those who have worked gold know that there is a great variety of behavior in gold, as in any other metal. Variety may be given by the manner in which it is treated. Pure gold is always the same in quality, but peculiar qualities are given to it by the method of treatment.

Dr. Brophy said that when gold was submitted to fumes of aqua ammonia it became non-cohesive. He had no choice in buying one rather than another. If he wanted it cohesive he annealed it; if he wanted it non-cohesive, he placed it in a drawer where it was exposed to the fumes of ammonia. He said that he had no experience in constructing bridgework, but he had examined the work of others. It claimed to be something strong and simple, supplying missing teeth without a plate, but what he had seen was anything but satisfactory in his judgment. For one or two teeth it might serve a very good purpose for a certain time, but for four or five teeth, say the first molar and two bicuspids even, it would soon result in failure. It was not possible to secure absolute adaptation of the teeth. There would sooner or later be a space in which secretions would accumulate and become exceedingly offensive, destroying the adjacent teeth. He would rather be without several teeth than wear such bridgework as he had seen. In the adaptation of incisor teeth there must be gold visible in bridgework. Why not have a gold plate? It would serve a better purpose. The bridgework would result in a few years in the loss of the teeth to which it was attached.

Dr. Horton, Cleveland, said that the present discussion of Operative Dentistry seemed to cover the whole field of practice. That it had been said that the old methods saved as large a proportion of teeth as was saved at the present time. He believed that, though the fathers had but little light, they worked up to the light they had; that the introduction of cohesive foil had added to the light, so that hundreds of teeth were saved now to one filled then. Teeth that formerly were doomed to the forceps are now saved. That Herbst did not stand alone in his method; that Blount gave his instruments a rotary motion, not rapid, but rotating sufficiently to carry the foil to the cervical walls. No man can succeed thoroughly until he has a pretty thorough knowledge of all methods. Combine the various systems with sound judgment; the head, hand and heart must all be educated in this work.

Dr. Thomas, Detroit. Dr. McKellops rather insinuated that I was not acquainted with the use of the mallet. I would say that I have Bonwill's mechanical and electric mallets and others, and use the mallet when necessary. Dr. McKellops said that proximal cavities could not be as well filled without the matrix as with it, but I do not believe he meant to say that.

Dr. Allport. For pits and retaining points I anneal one end of the pellet, but I put the unannealed end of every pellet down.

On motion the subject of Operative Dentistry, Section iv was passed.

A Communication was received from the Superintendent of Memorials for the Central Women's Christian Temperance Union, asking the adoption of the following resolutions:

*Resolved.* That we recognize and commend the excellent work done quietly but systematically by the Woman's Christian Temperance Union.

*Resolved.* That we endorse its movement for temperance education in the public schools; its proclamation against the use of alcoholic stimulants, and the efforts it is making to educate the people in regard to the laws of hygiene and heredity.

*Resolved.* That, as a body of practitioners, for the purpose of developing a moral feeling throughout the country, and as having a direct influence for good upon all classes, we will discourage the use of alcoholic stimulants in our practice.

Respectfully submitted.

Dr. Pierce, Philadelphia, moved the adoption of the resolutions.

Dr. Smith, Minneapolis, moved that they be so amended as to include the use of tobacco.

Dr. C. W. Spalding, St. Louis, moved that they be laid on the table.

Dr. W. C. Barrett, Buffalo, seconded the latter motion, saying that he did this, not from lack of sympathy with the cause represented, or with any intention of discourtesy, but because the matter is entirely foreign to the objects of the Association.

The motion to lay on the table was carried.

On motion adjourned to 3 p. m.

Thursday, 3 p. m.

The President in the chair.

On motion of Dr. A. W. Harlan, Surgery was added to Sec. vi, no special provision having been made heretofore for papers or discussions on the subject of Surgery.

On motion of Dr. Barrett, Sec. v was passed and Sec. vi called.

Dr. A. W. Harlan, Chicago, Chairman, reported a paper from Dr. J. D. Patterson, Kansas City, entitled "The Catarrhal Nature of Pyorrhea Alveolaris," and one from Dr. W. H. Atkinson, on Pyorrhea and Sponge Grafting; also, his own written report as Chairman of the Section.

Briefly summarized, Dr. Harlan said that there had been no discoveries in dental pathology during the past year, of sufficient importance to be placed on record, the most important being the researches of Miller, of Berlin, the vast import of which was not yet appreciated or understood. That two cases of actino microsis in the human subject, the fatal "lumpy jaw" of cattle had been reported, but that it did not appear to be serious, the second case even recovering without treatment; that the germs are constantly present in the mouth and throat and stomach, being readily found underneath the gum of erupting teeth, but were instantly destroyed by contact with oxygen; that they were not found in the pus from a fistulous outlet. He said that he had not been able to complete his report on Pyorrhea Alveolaris, and asked for further time. That it was important to extend the list of remedies, the most valuable and interesting drugs added during the past year being cocaine. His conclusion, from his own experience, was that cannabis indica was vastly more efficacious than any form of cocaine; that the former would obtund the most sensitive cavity, while painting the gums with the fluid extract renders them absolutely insensible to pain; an exposed pulp was anæsthetized in from five to ten minutes, that by saturating the gums, extraction was rendered painless when done with warm forceps dipped in the tincture. He had not had good results from cannabis indica in pyorrhea pockets. The antidotes were the same as for opium. Resorein and Terabin were new remedies as yet not generally appreciated by dentists. Resorein was a valuable substitute for carbolic acid, being neither poisonous nor escharotic, except internally. Terabin, transparent and limpid, and a ready absorbent of oxygen, is a stimulating disinfectant, antiseptic, and is especially useful in cases of closed abscess; it destroys all foul odors when used as a dressing for malodorous root canals. Has no obtunding qualities; is soluble in alcohol 1 to 10. Its merits are undoubted.

Dr. Patterson, Kansas, then read his paper entitled: "The Catarrhal Nature of Pyorrhea Alveolaris." This paper was an elaborate study of the comparative pathology of pyorrhea and catarrh. He said that catarrh was distinctively an inflammation of the air-passages, distinguished as nasal, pharyngeal, laryngeal, bronchial, etc. That the exudations changed from watery to pus and blood, the serous effusions being irritant and excoriating; that it was exceedingly infectious, adjoining parts being promptly affected. Its further stages were inflammation, destruction of function, formation of crusts, decomposition and ozæna, the formation of deep and penetrating nerves producing necrosis with bony deposits and osseous formations under the crusts. These catarrhal phenomena, affecting the cavities of the upper jaw, strongly resemble those of pyorrhea, in the inflammation of the gums, exudations of serum pus, and blood, the destruction of the ligaments of attachment, the deepening pockets, the calcareous deposits, and the absorption or necrosis of the septum and alveolus. Careful attention was directed to the similar pathology:

1. In the similar affection of the mucus membrane.

2. The identical character of the effusions, purulent serum, pus and blood.

3. Its infectious character, affecting adjoining teeth in pyorrhea as it does adjacent parts in catarrh.

4. The similar burrowing of pus.

5. The destruction of periosteum and bone.

6. The similar deposits, that of pyorrhea being familiar, the osseous deposits of catarrh being also fully described by authors, as Ziemssen's Medical Encyclopedia, where the chalky deposits and bony concretions of catarrh are described.

These similarities point to the conclusion that the two affections are similar in origin and in nature; that catarrh being a disease of the air-passages, and all tracts to the lungs being liable to contamination, pyorrhea is the result of local contamination from nasal drippings, or from poisonous sputa lodging around the teeth, or in case of mouth-breathers, originating in the gums as readily as in the nasal passages. Dr. Patterson said that he had made a special study of twenty-four cases; in many instances where catarrh was denied it was found present; that his observations had confirmed his theory in every case. The patient may be ignorant of and deny the presence of catarrh even when hypertrophic and fœtid symptoms are readily recognized by others.

Dr. Wm. H. Atkinson next read his paper entitled: "Pyorrhea and Sponge-Grafting," of which the following is an abstract:

He said that correct diagnosis was the first requisite; that there was no real pyorrhea without loss of attachment of the ligamentum dentium; a flow of pus was always an evidence of nature's failure to form new tissues. Pyorrhea was cured by destroying the microbes which prevent granulation; that an imperfect elimination of urea was an antecedent of pyorrhea; that traumatic pyorrhea was the resultant of the presence of foreign bodies in the alveolus, as particles of bone, splinters of wood, etc. These must be removed and the pockets sterilized. Calcareous deposits were not always present but if there must be removed and prevented. Many parisiticides which would destroy microbes would form schar-tissue, changing pabulum into cooked flesh. Nitrate of silver would afford protection by supplying an outer wall. Non-sterilized pockets might discharge pus for an indefinite length of time. There is a lack of knowledge of molecular changes. The object in writing was to state the results of experience.

He would prescribe tonics, as sulphate of quinine. For local treatment.

1. When there is slight recession, and no discharge, use Elixir Vitriol.

2. When the loss of attachment is greater, use Aqua Regia. 1 to 3.

3. When the loss is very great, the gums dark, with discharge of pus and bloody serum, use the carbolized potash paste, known as Robinson's Remedy, not allowing it to overflow on the lips or cheek.

4. When the roots are largely exposed and no opportunity of covering in the pockets to induce new growth, use the sponge-graft. This is a recent discovery, of which little generally is understood, but than which there is nothing of greater value. It grew out of the misfortune of traumatic lesions, having been first used by obstetricians to supply gaps caused by violent labor. The sponge is sterilized by treatment with bi-chloride of mercury; tents must be removed and changed, but the sponge remains permanently. We have advanced by successive steps from the actual cautery, boiling oil, red hot iron for the arrest of hemorrhage; then the irrigation and drainage of wounds; then transplanted skin and epithelial tissue. This suggested some material to take the place of lost substance; many forms of animal sutures and flesh tents were used, but these were generally extruded and cut off. It is not true, however, that chicken flesh, etc., was ever used to fill gaps. Sterilization or Listerism was a great step in advance. Then came the sponge graft. A gaping wound, filled with sterilized sponge and covered over with plaster to support and prevent exudation of blood and serum will heal by first intention if left undisturbed. All germs must be destroyed; there is a large list of germicides, but bi-chloride of mercury is the best.

Proper discrimination must be used in judging of the favorable or unfavorable progress of the healing process.

Dr. Abbott said that he was pleased to show the method of using his spray instrument for applying remedies in the mouth, with which they could be thrown wherever desired, etc. In abscess of the antrum, remedies could be applied directly to every point of the cavity. In case of hypertrophy there were more or less layers in the mucous membrane discharging pus; these should all be cleansed, the syringe could only wash off the surface, while this instrument would carry it in every direction, at the same time filling the cavity full. Dr Farrar had invented a syringe with similar points, but this was a more simple and more certain way of applying remedies, as the spray had intense force. In using remedies that were liable to crystalize, as the boracic acid or Listerine, water must be thrown through for cleansing after each application. The instrument is not confined to the use of Listerine, any liquid can be used. It has 60 lbs. pressure and is invaluable for the treatment of diseases of the antrum.

Dr. J. S. Marshall, Chicago, wished to ask Dr. Atkinson how to protect the graft after it was placed in position in the mouth ?

Dr. Atkinson. For the case of loss of substance by pyorrhea, after thorough removal of all deposits, tie loose teeth firmly, having the occlusion perfect; then take an impression and make a cast. Wherever the tissue is missing build it up with wax, rather higher than the new growth is wanted. Make a cast and strike up a plate, making a tight pocket, which will fit the teeth closely and prevent anything from escaping; put the sterilized sponge inside of this pocket. In larger places, as from removal of tumors or morbid growths, cover in with flaps of skin, leaving the clot of exudation. It does not form scar-tissue. It is reproduced tissue. Do not undertake to buy sterilized sponge; buy surgeon's fine sponge; see that it is free from all foreign elements; sterilize by dipping in a solution of 1 gr. bi-chloride of mercury to 1 oz. distilled water at 130° Far. At or above 133° will shrivel the sponge, and 160° will cook it so that it is not fit to use, as the living material that is designed to be absorbed is killed. If the sponge is not thoroughly sterilized it will not heal by first intention. Do not remove the sponge for any cause whatever; if pus should ooze from one corner, or the sponge get black or hard, where pabulum has not penetrated, wipe off the serum, and with fine pointed scissors cut off the discolored portions till red pabulum or arterial blood appears; sterilize the sponge again, fitting a little bit into the vacant space, not disturbing the adhering portions. Use simple common sense. Any man that can fill a tooth can be a good surgeon.

Dr. Marshall said that in his previous attempts the sponge would get displaced from the pyorrhea pockets, and he had almost concluded that the graft was useless. With Dr. Atkinson's method, as just described, he had no doubt of success in the future. He had tried Dr. Brigg's method of preparing the sponge by placing it in dilute hydrochloric acid, treating with ether and iodoform, but that the sponge dissolved and was good for nothing. In certain forms of perforation or cleft palate, he thought the sponge-graft might be practical and valuable.

Dr. Friedrichs, New Orleans, inquired if it would not be necessary to denude the surface?

Dr. Marshall. Of course, that has to be done in every case.

Dr. A. W. Harlan, Chicago, said that he had not been able to obtain as good results from cannabis indica as from cocaine; that the preparations of cannabis indica were very uncertain and he could get no two supplies alike; one would act beautifully; from another the results would be *nil.* Even from the samples given out that morning he could get no results on the tongue; it produced no effect on sensitive dentine but left a peculiar sensation in the teeth for thirty-six hours. He had had many cases of pyorrhea that he could not attribute to local causes, but that were probably of constitutional origin, as in patients of rheumatic or gouty diathesis, or suffering from glucosed urea, Bright's disease, etc. In such cases local treatment had been of no avail until the physician had mitigated the disease; then the pyorrhea was also better. The same was true in the case of ladies with uterine troubles. In these cases there would be pyorrhea without deposits, and all local treatment was vain until the patient was successfully treated by the Gynecologist, when there would be corresponding improvement in the mouth.

Dr. Davidson, Chicago, said where he had failed with hydrochlorate of cocaine he had succeeded perfectly with cannabis indica. He described a case of caries of the lower central incisor, running to the margin of the gum and very sensitive; he applied the dam, and cannabis indica, and with the engine removes a large portion of dentine without any complaint from the patient. On the first symptom of pain he again applied the remedy, working on another tooth for a few moments; when he returned to the central incisor without warning to the patient, it was all right again.

Dr. F. Abbott, N. Y., said that his Atomizer was valuable for blowing chips from the cavity, as it gave a continuous stream of air; that it was also excellent for local anæsthetizing; the tooth being rendered insensible to pain in a moment's time.

Dr. Taft suggested its use with the warm air blow pipe.

Dr. Abbot replied that an alcoholic lamp or gas jet could be introduced underneath for keeping the air heated; heating compressed air not increasing its force, even if raised to 120° or 140°.

Dr. Taft, said that 200° would increase its intensity; it would be well to advise caution--not to go above a certain temperature.

Dr. Abbott said it would never be necessary to go above the heat of the body.

Dr. Thompson, Topeka, said he had a case of Pyorrhea in a patient whose habits were not cleanly, and his teeth very bad; he had also a very offensive case of catarrh, but had never had any treatment. He prescribed preliminary treatment for the catarrh, with salt water douche, etc. The improvement in the catarrh resulted in marked improvement of the pyorrhea also. The blending in pyorrhea of most of the symptoms of catarrh, suggested that when pyorrhea was present there was also nasal catarrh, and he found this invariably confirmed by the physician. He had investigated, after the suggestion of its renal origin, but could not trace it to that source. He said that Dr. J. G. Templeton claimed wonderful results from the application of copper sulphate (blue stone) in the pockets once a week. That he had had considerable experience in the use of cocaine, meeting with some success and some failures; success in, perhaps, 60 or 70 per cent., but that it was impracticable in many cases, requiring too much time to produce its results. Neither he nor his patients could afford to wait twenty minutes, and then twenty more.

By taking full time the desired result would, doubtless, always be secured; it was reliable, but the time required made it impracticable.

Dr. King, Lincoln, Nebraska, said that he had some experience and some success in the treatment of pyorrhea; that having kept a record of the history for a large number of cases, with the constitutional history of each patient, his experience failed to confirm the theory that the disease was due to an impoverished condition of the blood; at least 50 per cent. being people in good health, with robust, vigorous constitution. He considered that calcareous deposits were the immediate cause of the disease, though there was, of course, something back to cause the deposits. If this was not so, then why was it so essential to remove all deposits? Why insist on keeping the teeth free from subsequent deposits to prevent the return of the disease? In cases where no salivary deposits are present, it will always be found on tracing the case back, that it had been the initial trouble, though the system had so changed more recently that the deposits were not continued.

Dr. Ingersoll wished to correct a statement which had recently appeared in the Journals, as quoted from Dr. Rawl's paper on Pyorrhea, where he (Ingersoll) was represented as saying that the gathering of salivary calculus was the cause; that he had distinctly guarded that point; that it was never the cause, but uniformly the result.

Dr. Friedrichs wished to inquire whether there were any recognized cases of calculus without pyorrhea. He had one case where there were considerable deposits which the patient would not allow touched, and, which, he said, had been unchanged for twenty-five years. The gums were of healthy, natural color; no pus, no inflammation, the alveolus very thin.

On motion, the regular order of business was suspended. The

Chairman of the Local Committee made some announcements regarding the excursions.

Adjourned to 8 p. m.

Thursday, 8 p. m.

Called to order; the President in the chair.

Dr. Kulp, Iowa, asked for information concerning the Section of Dental and Oral Surgery of the International Medical Congress.

Dr. Allport, Chairman of the Committee, replied

After some discussion the subject was postponed.

A letter was read from Dr. Clifton, of Waco, Texas, thanking the members of the Association for kind attention and sympathy at the time of the accident to his little son during the Saratoga meeting.

On motion, Sec. VI was passed. Sec. I called. Dr. Wm. H. Trueman, Secretary, read a paper on a "New method of flasking vulcanite rubber work," a method which offers advantages in cleanliness, saving of time, and safety of teeth, especially in difficult partial cases. By the original English method, the work was invested in steatite or soapstone. Dr. J. Spyer, of Philadelphia, has modified the method, using the patented "Surface Cohesive Forms," of prepared tin-foil, similar to that used for stipple work, or for securing a clean, bright, palatal surface. The forms are embossed with geometrical figures which leave a network of continuous channels instead of a central suction cavity. This gives firmer adhesion, causes less irritation to the mucuous surface and allows a much smaller plate, even if the plate tilts the suction not being entirely lost. The portion of the model to be covered with the *form* is thickly painted with a cement made of vulcanizable rubber dissolved in chloroform to the thickness of a heavy syrup, the foil being pressed down till all the indentations are filled with the cement, which prevents crushing out of shape in packing. Rubber softened in hot water is used in mounting the teeth instead of wax or gutta percha base plates, small bits of softened rubber, and the cement being packed in around the teeth and pins. The rubber is built up to the proper size and shape and finished as wax work is generally used. It is then invested in plaster, without allowance for opening the flask, as there is no wax to be removed, and the cover screwed down before the plaster sets, and it is ready for the vulcanizer. While time and labor are saved, and cleanliness insured, the method has the great disadvantage of not allowing of trial in the mouth until finished.

The rubber dissolved in chloroform forms a cement which is valuable for many purposes, as for repairing bulbs of syringes, etc.

Dr. Haskell, Chicago, read a paper entitled, "What to do and how to do it." He said that many dentists did not appear to appreciate the vast difference in mouths. A patient will ask: "Why are not my teeth as easily made satisfactory as those of Mrs. ———?" They evidently think the difference lies in the dentist, but it is more probably due to the difference in mouths. As we all know, some mouths are very easy to fit, while others require great care and skill and study, and the patient requires great patience in learning to wear the plate. Where there is a firm ridge, not deep, the palatal surface not hard, the lower jaw not protruding but striking fairly under, there will be no trouble in putting in a satisfactory plate. But the contrary may be the case in some or all points, as where there has been great absorption, with flexible ridge—as we find to a fearful extent under vulcanized rubber plates by which thousands of mouths have been ruined, the better the adaptation the worse being the results. When the plate is loose-fitting less damage is done; where the suction is perfect, an abnormal condition prevails from undue retention of heat. It is not correct to say that the process is absorbed; it is simply that waste material is not replaced. For this reason rubber plates are doing incalculable damage; the process is often entirely gone and the plate resting on the muscles and soft tissues, skill must be used in fitting the plate and in antagonizing the teeth. Many plates are apparently worthless, simply from faulty antagonizing, which a little skillful grinding would make quite useful and comfortable. All conditions of the mouth are found ranging between the two extremes. In preparing the mouth for artificial teeth the extraction of the remaining teeth is a point requiring especial consideration. While it is true that it is the provision of the dentist to save the natural teeth, when the time has come that the patient must wear some artificial teeth, the question arises: shall it be a full or a partial set. When the former involves the extraction of some remaining teeth, a safe rule to follow is to remove whatever will interfere with the comfort and utility of the plate, throwing aside all sentiment regarding sound teeth if they are in the way of making the plate completely comfortable and useful. As to the material for the plate, rubber is universally used the world over, because it is cheap and easy. Any young man can learn to make a rubber plate in a few weeks, in a few weeks more he learns to insert amalgam fillings in simple cavities, and this constitutes the whole stock in trade of many who call themselves dentists. Metals being good conductors of heat are preferable in the mouth. When undue absorption takes place under a metal plate, as is sometimes the case, it will be found that there has been undue pressure in front, but this is exceptional.

Whenever it is possible, a patient should be induced to have a metal plate. You say you have no demand for metal plates. You must create the demand. Ask the average patient what kind of a plate he wants, and as a rule he will answer, *rubber*. Ask him why he wants rubber and you will find that he seldom has any better reason than because all his friends wear it. Explain to him that it is not the best, and why it is not the best though the cheapest; he will probably understand that the cheapest is not the best in teeth any more than in clothing, though it is true that many who want only the very best that money can buy in clothing, will go shopping to find the cheapest in dentistry. If you cannot make anything but rubber, if, though having been educated in the manipulation of metal, having had no demand for it, you have never practiced using it, and have forgotten what you once did know about it, it is an unfortunate state of affairs.

For taking impressions, various substitutes for plaster are being used, modeling, compound wax, etc.; but for a perfectly correct impression, plaster is the only material that is absolutely reliable, and everything depends upon a correct impression. I use no air-chambers, but swage the plate right up to the palate in every case. I recently saw a number of old patients who were wearing plates that I put in twenty or twenty-five years ago; in every case they were swaged right up to the palate, and the alveolar process was just as solid and the mouth as healthy as the day they were put in. But of late years we find many mouths in which the process is entirely gone, and only flexible membrane left. I raise the cast slightly in the centre with a thin film of wax, scraping out a little when necessary to make the plate set snug in the soft parts. There is but one metal that possesses all the requisits for dies, and that is genuine Babbitt's metal. It has the necessary hardness, smoothness, and toughness, but you must be sure that it is Babbitt's metal. If it is offered for twenty-five or thirty cents, it is worthless, lead has been substituted for tin, which makes it too soft; that made by S. S. White or Justi is good. The formula is 1 copper, 2 antimony, and 8 tin. For the counter-die, $\frac{1}{8}$ tin must be added to the pure lead or it will soften and adhere. You will seldom have to make a second die, if your materials are right. One die will swage it to fit accurately. When the die fits the plaster cast, it will also fit the mouth. Dr. Haskell said that he considered that bridgework, or the system of teeth without plates, was doing incalculable injury. It was impossible that a plate permanently attached to the natural teeth should be otherwise than uncleanly. If placed under the nose when removed for repairs it would be found absolutely disgusting. The teeth to which the gold band of the bridgework was attached with cement must inevitably loosen and disintegrate. It was impossible to prevent the secretions of the mouth from going there to stay, and the teeth of attachment would soon be girdled with decay, and loosened in the jaw.

The method exhibited by Dr. Parmly Brown was the least objectionable, and might be admissable in some cases, but even that might be carried to extremes, and a long bridge would inevitably go at last. There was no necessity for it except occasionally as a last resort, but in the majority of cases a narrow gold plate, with properly adjusted clasps, which could readily be removed and kept clean, would answer every purpose, and would do no harm and occasion no strain on the natural teeth.

Dr. Ames, Chicago, said there were but two methods of retaining upper plates; spiral springs, which were no longer used, and atmospheric pressure. The usual form of a large chamber over the centre of the palate afforded unequal support, while the tissues soon filled the cavity. The plate should be so constructed as to equalize the bearing, atmospheric pressure being applied just where needed. Trimming the cast was not sufficient, but the plate must extend to a point where the edges will displace the soft tissues, running well up beneath the lip and cheeks so they will lie snugly on the edge. Instead of raising the centre, as described by Dr. Haskell, would trim away where soft, having a groove across the portion of the plate where the tissues are lax. If this cannot be tolerated because of nausea, would use soft rubber edge.

Dr. Wm. H. Morgan wished to notice one or two fundamental errors. Did not appear as the champion of rubber, which was a poor article for base, causing troubles which would not occur with a gold plate. The first speaker from Chicago left the inference that the damage done by rubber was due to undue heat; this was a mistake; the mucous membrane does not eliminate heat; it could not get above blood-heat unless there was inflammation; it could not eliminate enough heat to raise the temperature of the mouth above that of the body. The idea of undue heat was a faulty notion; there is always irritation and inflammation preceding abnormal heat. We must go back of that for the production of loose tissues in the front of the mouth. Again it was said that all the natural teeth should be removed that would interfere with the artificial denture—the contrary should be the rule; the comfort and utility of the natural teeth should be considered, and the plate made to conform to them.

Dr. Horton, Cleveland, asked Dr. Morgan to explain the production of the loose tissues.

Dr. Morgan. We don't know; it is true that it is worse under rubber plates, but it is not from undue heat.

Dr. Atkinson. The gentlemen who so unqualifiedly condemn bridgework have not seen it properly constructed, or they would speak differently. Perfect bridgework, intelligently attached, is as cleanly as anything possibly can be. They don't know that an arm can be put into the tooth and make it so comfortable that the wearer sings hallelujah! Rubber first anæsthetizes the sensitive membranes, and then paralyzes the sensory nerves. it allows the blood to flow in and form little lakes, destroying the lime-salts. If we knew what we were about, we would have no difficulty with shopping-people in our offices. I want my patients to regard me as an honest man with a conscience. I say to them: "I will do the clean thing by you, as I am an honest man. Love me well enough to believe that I will do what is right." People don't want nasty things in their mouths, and rubber is nasty beyond all things.

Dr. Haskell, Chicago. Dr. Atkinson has answered Dr. Morgan admirably. As regards bridgework, I excepted the method advocated by Dr. Atkinson, as practiced by Dr. E. Parmly Brown, as being cleanly, if properly inserted.

Dr. Morgan (to Dr. Atkinson). Did you say there could be heat above the normal, without irritation?

Dr. Atkinson. No; there is always irritation and inflammation under a rubber plate.

Dr. C. W. Spalding. If you will take the trouble to test the actual temperature under a rubber plate and under a gold plate, you will not find 4° difference. Anyone can make the test and satisfy himself.

Dr. Brophy, Chicago. It was well said that the tissue becomes inflamed under rubber plates. Why? Because the vegetable substitutes not being good conductors, cold contracts the capillaries around the plate, but not beneath it; the blood rushes to the capillaries beneath, and forms little lakes of blood. Then follows congestion and absorption; not because over-heated under the plate, but because the surroundings are of unequal or uneven temperature. The inequality of temperature beneath and around the plate causes the trouble.

Dr. C. W. Spalding. The irritation arises largely from roughness of surfaces.

Dr. W. C. Barrett. Dr. Spalding will find that if the inner surface is polished, the inflammation will be increased.

Dr. Morgan. I find that large numbers get well when polished.

Dr. Atkinson. The closing of the mucous follicles will produce mischief. If the plate is worn during sleeping hours, the secretions will be changed to pus. I have seen it all over the roof of the mouth. I knew a dentist who wore celluloid; knew it to be A No. 1. I looked into his mouth and found it to be all over apthous patches, due to paralysis of the sensory nerves. He was cured in six months by wearing a metal plate. No plate should be worn all night.

Dr. Spalding. In reply to Dr. Barrett, I would say that when inflammation is increased on polishing a plate it is because the fit of the plate has altered.

Subject passed.

Adjourned to Friday, 9 A. M.

FRIDAY, 9 A. M.

The President in the chair.

Minutes read and approved.

Dr. Dudley moved that the Chair appoint a committee of seven to consider the feasibility of holding an international dental congress in 1887. Said committee to correspond with the various dental bodies and leading men in the profession, and report at the next meeting of the Association.

Dr. Barrett said that the matter required careful consideration.

The motion was carried without discussion.

The Chair appointed:

Dr. Dudley, Salem, Mass., Chairman.

Dr. C. N. Pierce, Philadelphia.

Dr. Frank Abbott, New York.

Dr. M. W. Foster, Baltimore.

Dr. Brophy, Chicago.

Dr. Thompson, Topeka.

Dr. Bauer, New Orleans.

Section VII reported as subjects for investigation during the ensuing year:

"Normal and Abnormal Oral Fluids."

"The Tongue in Health and in Disease."

"The Personal Hygiene of Dentists."

On motion of Dr. Harlan, a vote of thanks was tendered the railroads, steamboats, and hotels; the press of Minneapolis and St. Paul; the dental societies of Minnesota and Minneapolis, and the local committee.

Thanks were also returned to the Trustees of the Curtis Commercial College, where the meetings of the Association were held.

After some further announcements relative to the proposed excursions to Lake Minnetonka, the flouring mills, and the Yellowstone Park.

Dr. E. Parmly Brown read a short paper entitled, "Dentistry: its Past; its Encouraging Present; its Brilliant Future." He said that dentistry, once looked upon as almost vulgar by the masses, had been hybridized with truth, impregnated with learning, illuminated with art, and stimulated by poetry. That while medicine was almost a pure science, and surgery an artistic science, dentistry was a scientific art, strictly artistic in the execution of its practice, and scientific in the preparation of the workman for his work. That dentistry was not a part of any thing, but a something complete within itself, closely allied to medicine and surgery, but more operative and more artistic than either. That dentistry, while in no sense a specialty of medicine, yet had its own specialties, as dental surgery, operative dentistry, prosthetic dentistry, anæsthetic dentistry, regulating dentistry, etc.—more than any one brain and body could master in all its details. The successful dentist of the future must be an educated and polished gentleman in addition to being master of his profession. That the lady of the future would select her dentist with ten times more fastidiousness than she will her physician. That the dentist must choose wisely between rusting out and wearing out, to make sure of holding out. It was violating the laws of physiology, philosophy, and christianity to labor unceasingly, and transmitting an evil influence to posterity. There is no more wisdom in overwork than in making oneself miserable in trying to be happy.

The dentist who tries to do without sunlight will be but a sickly plant, a stunted tree. The growth of dentistry had been like the progress of the means of illumination—beginning with the ignition of a pine-knot with the spark from a flint; the moulded tallow candle; the friction-match and illumination by gas; and finally the dazzling light of electricity. Comparable to the latter are the microscopic investigations, and the ingenious appliances of the dental profession of the present. Ideal dentistry will keep pace with all other things good for man's advancement. An appreciative public creating a demand for it will produce it.

The next order of business was the selection of the next place of meeting. The Executive Committee reported that they had received invitations from Louisville, St. Louis, Detroit, New Orleans, Deer Park, Oakland, Niagara, Newport, R. I., Crescent Springs, Pa., Lake George, Asbury Park, N. Y., and other places.

Dr. Atkinson moved that the matter be referred to the Executive Committee, with power to confer with railroads, hotels, etc., and select the place of meeting, the same to be announced through the journals.

The constitution requiring that the place be chosen by ballot, the rules were suspended, and the motion carried unanimously.

The next order of business was the election of officers. Of 119 votes cast on the first informal ballot for President, Dr. W. C. Barrett, Buffalo, received 61, Dr. M. W. Foster 43; the rest scattering. The ballot was made formal, and Dr. Barrett declared elected President for the ensuing year.

Dr. Ingersoll was elected 1st Vice-President.

Dr. A. F. Smith, Minneapolis, 2d Vice-President.

Dr. Geo. H. Cushing, Recording Secretary, re-elected.

Dr. A. W. Harlan, Corresponding Secretary, re-elected.

Dr. Geo. W. Keely. Treasurer, re-elected.

Dr. Thompson offered the following resolution:

"*Resolved*, That as an expression of the appreciation of the valuable services of Dr. J. N. Crouse, President of this Association for the closing year, and of his many excellent personal and professional qualities, he be presented with the gavel which he has so effectively wielded, as a souvenir of this most successful and enjoyable meeting, and the same be appropriately mounted and inscribed."

Unanimously adopted.

Dr. Pierce offered an amendment to the constitution, vesting the choice of place of meeting in the Executive Committee. Laid over for consideration.

The Committee on Necrology reported the following resolutions:

IN MEMORIAM.

"WHEREAS, This Association has received intelligence of the death of Dr. Isaiah Forbes, of St. Louis, Mo., who has long been identified with the interests of the dental profession in this country; and

"WHEREAS, This Association, as an expression of the high esteem in which the deceased was held, adopt the following:

"*Resolved*, That in his death the profession of dentistry has lost a careful, conscientious, and ever-faithful member; one who was justly entitled to the respect and regard of his associates throughout his long professional career. For a number of years he creditably filled a chair in the faculty of the Missouri Dental College, and was at all times active and earnest in all laudable efforts for the promotion of the higher interests of the profession of which he was a member.

"*Resolved*, That a copy of this preamble and resolution be properly engrossed and sent to the family of the deceased, and also copies be sent to the dental journals for publication."

"WHEREAS, information has been received by this Association of the death of Dr. J. G. Ambler, of New York, who has long been an active member of this Association, and who, for more than forty years occupied a prominent position in the dental profession; he was ever ready to do what he could for the promotion and elevation of his chosen profession. Therefore,

"*Resolved*, That in his death the profession has sustained a loss which is sincerely deplored, and this Association a valuable member, who was active for the promotion of its best interests and prosperity, and that we regard his social and professional qualities as deserving high esteem, and his example as worthy of imitation.

"*Resolved*, That a copy of this preamble and resolutions be sent in proper form to the family of the deceased, and also to the dental journals for publication."

"While we humbly bow to the inevitable decree of the Disposer of all things, in the removal of General Grant, this Association would record its high appreciation of his public service, its deep sense of loss, and its sincere sympathy with his family and friends, and with our countrymen at large.

"*Resolved*, That a copy of this resolution be sent to the journals for publication." "J. TAFT,

"C. W. SPALDING,

"W. H. ATKINSON,

"*Committee*."

Dr. Hunt, from the Section on Dental Education, reported, approving the action of the Association of College Professors with regard to improving the literature of the profession, its text books, etc. Also, their action with reference to equivalent courses in medical colleges, diploma of colleges, on high school, and other points.

The report was accepted.

Drs. Field and M. A. Morrison then conducted the President elect to the chair. The retiring President presented him with the battered and broken gavel and asked for his successor the same kind consideration he himself had received.

Dr. Barrett accepted the office with a few brief but appropriate words. He thanked the Association for the great and unsolicited honor, saying that he would be either more or less than human if he failed to appreciate the compliment paid; that he loved dentists more than any other class of men in the world.

The President was instructed to appoint a Local Committee as soon as the place of meeting should be announced.

The Secretary, Dr. Cushing, Dr. A. W. Harlan, and Dr. E. T. Darby, were appointed Committee on Publication.

The gavel, which he had wielded not only with discretion and grace, but also with such force that it was battered and broken, was presented to the retiring President, with instructions that it be repaired and suitably inscribed.

With the hope that the next meeting would be as pleasant and as successful and as many new members enrolled as on the present occasion, at 12 m. the Association adjourned *sine die.*

### THE EXCURSION.

At 2 p. m., the members, accompanied by a large number of ladies and invited guests, assembled for the last time at Curtis' Hall and proceeded to the depot of the Minneapolis & St. Louis R. R., where a special train of seven coaches was awaiting to convey them to Lake Minnetonka. Boarding the fine steamer, the Belle of Minnetonka, they made the tour of the lovely lake, touching at Excelsior, Hotel St. Louis, and other points. The weather was delightful; the sunset gorgeous, and twilight deepened into darkness ere the Lake Park Hotel was reached, where an elegant dinner awaited them.

Numerous toasts were proposed and responded to; Dr. A. W. Spalding, of Minneapolis, acting as Toast Master. To the toasts characteristic responses were made by a number of the members. The Committee of Arrangements had done their duty so perfectly that the occasion was one of unmixed pleasure. The next morning, by special invitation, a large party visited the celebrated flouring mills of Minneapolis. Many visited the lovely Falls of Minnehaha, noted in poetry and song. Others spent the day at St. Paul. On Saturday quite a numerous party left for the Yellowstone Park. The last lingerers turned their faces homeward, and the 25th Annual Session of the American Dental Association became a thing of the past, long to be pleasantly remembered.

